# SLC30A3 (ZnT3) Oligomerization by Dityrosine Bonds Regulates Its Subcellular Localization and Metal Transport Capacity

**DOI:** 10.1371/journal.pone.0005896

**Published:** 2009-06-12

**Authors:** Gloria Salazar, Juan M. Falcon-Perez, Robert Harrison, Victor Faundez

**Affiliations:** 1 Divison of Cardiology, Department of Medicine, Emory University School of Medicine, Atlanta, Georgia, United States of America; 2 Department of Cell Biology, Emory University School of Medicine, Atlanta, Georgia, United States of America; 3 Metabolomics Unit, CIC bioGUNE, CIBERehd, Bizkaia, Spain; 4 Department of Computer Science, Georgia State University, Atlanta, Georgia, United States of America; Harvard Medical School, United States of America

## Abstract

Non-covalent and covalent homo-oligomerization of membrane proteins regulates their subcellular localization and function. Here, we described a novel oligomerization mechanism affecting solute carrier family 30 member 3/zinc transporter 3 (SLC30A3/ZnT3). Oligomerization was mediated by intermolecular covalent dityrosine bonds. Using mutagenized ZnT3 expressed in PC12 cells, we identified two critical tyrosine residues necessary for dityrosine-mediated ZnT3 oligomerization. ZnT3 carrying the Y372F mutation prevented ZnT3 oligomerization, decreased ZnT3 targeting to synaptic-like microvesicles (SLMVs), and decreased resistance to zinc toxicity. Strikingly, ZnT3 harboring the Y357F mutation behaved as a “gain-of-function” mutant as it displayed increased ZnT3 oligomerization, targeting to SLMVs, and increased resistance to zinc toxicity. Single and double tyrosine ZnT3 mutants indicate that the predominant dimeric species is formed between tyrosine 357 and 372. ZnT3 tyrosine dimerization was detected under normal conditions and it was enhanced by oxidative stress. Covalent species were also detected in other SLC30A zinc transporters localized in different subcellular compartments. These results indicate that covalent tyrosine dimerization of a SLC30A family member modulates its subcellular localization and zinc transport capacity. We propose that dityrosine-dependent membrane protein oligomerization may regulate the function of diverse membrane protein in normal and disease states.

## Introduction

The quaternary structure of polytopic transmembrane proteins plays a critical role in defining their subcellular localization and, consequently, their function. For example, homo-dimerization regulates function and trafficking along the exocytic pathway of proteins as diverse as neurotransmitter transporters [1], cell adhesion molecules [2], G-coupled protein receptors [3] and membrane proteins in the ER-Golgi intermediate compartment [4]. Similarly, homo-oligomerization regulates the subcellular distribution and signaling capacity of receptors in the endocytic pathway. These include GABA, leukotriene B(4), and Toll/interleukin-1 receptors [5]–[7]. Critical to understand the effects that membrane protein homo-oligomerization exert on proteins is the definition of chemical interactions that hold membrane protein homo-oligomers. Identification of key residues and interfacial domains offers molecular targets to assess the functional role of chemical modifications involved in oligomerization and to predict homo-oligomerization in other membrane proteins. Here we present a new covalent homo-oligomerization mechanism in a member of the SLC30A family of zinc transporters that depends on redox-regulated covalent tyrosine dimerization.

Homo-oligomerization of membrane proteins occurs through non-covalent and covalent interactions, primarily within transmembrane domains. These interactions rely on glycine, leucine or cysteine residues. Among the non-covalent interactions, the most common involve GxxxG and GxxxG-like domains such as those found in glycophorin A, membrane transporters, and receptors [8], [9]. On the other hand, covalent oligomers are mostly mediated by disulfide bonds, like those in cell adhesion molecules and signaling receptors [2], [10]. A far less explored covalent oligomerization mechanism is that dependent on dityrosine bond formation. Dityrosine bonds are present in a limited group of structural proteins of the bacteria cell wall, invertebrate connective tissue, and in proteins of the vertebrate extracellular matrix [11]–[14]. Dityrosine bonds have been found in only one membrane protein, the angiotensin II AT2 receptor [15]. Dityrosine bond formation increases with aging, cellular stress, UV and γ irradiation and disease [16], [17]. Increased levels of dityrosine modified proteins have been found in lesions such as atheromatous plates [18] and cataracts [16]; in pathological processes such as acute inflammation and systemic bacterial infection [19]. Recently dityrosine bonds have been associated with α-synuclein fibrillogenesis [20] and Aβ amyloid oligomerization [21]. In all these cases dityrosine bonds are thought to represent the cumulative damage of a protein or to regulate protein function by either decreasing the solubility of secreted proteins or increasing oligomer resilience to mechanical stress.

In this study we show that dityrosine bonds induced the oligomerization of the zinc transporter 3 (ZnT3), a member of the SLC30A family of zinc transporters. SLC30A family members reduce the cytoplasmatic concentrations of free zinc, either by mediating zinc efflux from the cell or by metal influx into intracellular compartments [22]. One of the most studied members of this family is the zinc transporter 3 (ZnT3). ZnT3 is expressed in brain where it transports zinc into synaptic vesicles [23]. Deficiencies in either ZnT3 (ZnT3^−/−^) [24] or in the machinery that regulates its subcellular localization [25], [26] severely deplete synaptic vesicles zinc content. Despite the relevance of this transporter to synaptic physiology, mechanisms regulating SLC30A members zinc transport activity remain poorly explored. This is due to a lack of structural information, which has been fundamental in the elucidation of regulatory mechanisms in other type of transporters, such as chloride channels [27]. The existence of ZnT oligomers has been suggested for ZnT1 and ZnT5, 6 and 7 [28]–[31] and recently for ZnT8 [32]. Moreover, the crystal structure of the bacterial SLC30A3 homolog, Yiip, revealed non-covalent dimers [33]. Here we show that in contrast with YiiP, ZnT3 forms covalent dimers mediated by intermolecular dityrosine bonds. Dityrosine bond formation occurs both spontaneously and induced by oxidative stress. Using site-directed mutagenesis we identified two critical tyrosines in the carboxy-terminal of ZnT3, one that prevented and one that increased dimerization. Our studies indicate that covalent tyrosine dimerization of a SLC30A family member modulates its subcellular localization and zinc transport capacity.

## Materials and Methods

### Antibodies

The following antibodies were used for western blots: monoclonal anti transferrin receptor (H68.4), rabbit polyclonal anti SUMO2/3 and monoclonal anti SUMO1 were from Zymed Laboratories/Invitrogen (Carlsbad, CA). Ubiquitin monoclonal antibody was from Covance (Berkeley, CA). Anti SV2 (10H4) was from the Developmental Studies Hybridoma Bank (University of Iowa, Iowa City, IA). Monoclonal anti synatophysin (Sy38) was from Chemicon International/Millipore, (Billerica, MA). Vamp2 (69.1) antibody was from Synaptic System (Goettingen, Germany). Polyclonal antibodies anti myc and HA were from Bethyl Laboratories, Inc (Montgomery, TX). Monoclonal anti-β-actin (clone AC-15) was from Sigma (St Louis, MO). Polyclonal anti acetylated lysine was from Cell Signaling Technology, Inc. (Danvers, MA). Affinity purified polyclonal antibodies against PI4KIIα were described by Guo et al [34]. The following antibodies have been described previously: polyclonal anti ZnT3 [26] and mouse anti Vamp7-TI [35].

### Cell culture and drug treatments

HEK293T cells were cultured in DMEM medium (Cellgro, Herndon, VA; 4.5 g/l glucose) containing 10% FBS (Hyclone, Lolgan, UT), 100 U/ml penicillin and 100 mg/ml streptomycin. PC12 cells were cultured in the same media, but with 5% FBS and 10% Horse serum. Transfected cells lines were maintained in media containing 0.2 mg/ml G418 as described previously [36]. Incubations with H_2_O_2_ or drugs were performed in RPMI medium at 37°C. Cells were incubated with H_2_O_2_ (Sigma) during 20 min to 1 h or MG-132 (Calbiochem) 10 µM during 12 hrs. Catalase 500 U/ml or the ROS scavengers N-acetyl cysteine (NAC, Sigma) 10 mM or EUK-134 50 µM (Cayman Chemical, Ann Arbor, MI) were added to cells 15 min prior to the addition of H_2_O_2_ 10 mM and maintained during the H_2_O_2_ incubation. Myc-tagged zinc transporters ZnT4, ZnT5 and ZnT7 were expressed in HEK293T cells due to their low expression levels in PC12 cells.

### DNA constructs

pcDNA3 vector (Invitrogen) containing the human amino terminal HA tag SUMO1, SUMO2 and SUMO3 constructs were obtain from Dr. John Hepler, Emory University. pCR3.1 vectors (Invitrogen) containing human zinc transporters ZnT1, ZnT3, ZnT4, ZnT5 and ZnT7-myc tagged in the carboxy-terminal domain were previously described [37].

### Site directed mutagenesis chimeras construction and DNA transfection

All mutants were created using the QuickChange mutagenesis kit (Stratagene, La Jolla, CA). Oligonucleotides for tyrosine to phenylalanine mutagenesis of the human ZnT3 were the following:

Y330F mutant: sense-5′-GCCCTTACGCTCACTTTCCATGTTGCCTCTGCAC-3′ and antisense-5′-GTGCAGAGGCAACATGGAAAGTGAGCGTAAGGGC-3′.

Y357F mutant: sense-5′-CTGAAGCCTCATCCCGGCTCTTCTCCCG-3′ and antisense-5′-CGGGAGAAGAGC CGGGATGAGGCTTCAG-3′.

Y372F mutant: sense-5′-GCAGGTCGAGCAGTTTCAGCCG GAGATG-3′ and antisense-5′-CATCTCCGGCTGAAACTGCTCGACCTGC-3′.

Oligonucleotides for tyrosine to phenylalanine mutagenesis of the human ZnT4 were the following:

Y355F mutant: sense-5′ ATGAAAATAGAAGATGTATTTTCAGTCGAAGATTTAAAT-3′ and antisense-5′-ATTTAAATCTTCGACTGAAAATACATCTTCTATTTTCAT-3′.

Y404F mutant: sense-5′-TTGAACACATTTGGCATGTTTAGATGTACTATTCAGCTT-3′ and antisense-5′-AAGCTGAATAGTACATCTAAACATGCCAAATGTGTTCAA-3′.

Y413F mutant: sense-5′-ATTCAGCTTCAGAGTTTCAGGCAAGAAGTGGAC-3′ and antisense-5′-GTCCACTTCTTGCCTGAAACTCTGAAGCTGAAT-3′.

Human ZnT3-myc chimera (mhZnT3-myc) containing the amino terminal domain of mouse ZnT3 was constructed by adding an EcoR1 restriction site using the following oligonucleotides:

Mouse N-Terminal (1–252): sense-5′-CACCATGGGAGCCTTCTCTGGCCACC-3′ and antisense-EcoR1 5′-GAATTCGAAGCACACAGCGCAGGC-3′.

Human (259–1167) sense EcoR1 5′-CACCGAATTCATGGCTGGGGAGG-3′ and antisense myc 5′-TCACAGATCTTCTTCAGAAATAAGTTTTTGTTC-3′.

The addition of the EcoR1 site create an amino acid change from V85 to E that was corrected by site directed mutagenesis using the following oligonucleotides:

Sense-5′-GCTGTGTGCTTCGTATTCATGGCTGGGG and antisense-5′-CCCCAGCCATGAATACGAAGCACACAGC.

Mouse ZnT3-HA chimera (hmZnT3-HA) containing the amino terminal domain of human ZnT3 was constructed by adding an EcoR1 restriction site using the following oligonucleotides:

Human N-terminal (1–252): sense-5′-CACCATGGAGCCCTCTC-3′ and antisense-EcoR1 5′-GAATTCAAAGCAAACGGCACA-3′.

Mouse (259–1167) sense EcoR1 5′-CACCGAATTCATGGCCGGGGAG-3′ and antisense HA 5′-TCAAGCGTAGTCTGGGACGTCGTA-3′. The amino acid E added by the creation of the EcoR1 site was corrected to V by site directed mutagenesis using the following oligonucleotides:

Sense-5′-GTGCCGTTTGCTTTGTATTCATGGCTGGGGAG and antisense-5′-CTCCCCAGCCATGAATACAAAGCAAACGGCAC-3′.

Oligonucleotides were from Sigma Genosys. All mutants and constructions were evaluated by DNA sequence. 1 µg of DNA was transfected in PC12 or HEK293 cells using 4 µl of Lipofectamin 2000 during 16 hrs. After 48 hrs, transfected cells were selected with 0.8 mg/ml G418 during one week. Mouse ZnT3-HA has been previously described [26].

### Cross-linking and immunoprecipitation

Cells were washed twice in cold PBS plus CaCl_2_ and MgCl_2_ (PBS-CM) and incubated with 1 mM DSP (Pierce, Rockford, IL) or DMSO alone in PBS-CM for 2 hrs on ice as described [38]. The reaction was stopped by adding 25 mM Tris pH 7.4 for 15 min on ice and washing twice in PBS. Cells were lysed 30 min on ice in buffer A (150 mM NaCl, 10 mM HEPES, 1 mM EGTA and 0.1 mM MgCl_2_, pH 7.4), 0.5% Triton X-100 plus Complete™ anti-protease mixture (Roche Molecular Biochemical, Indianapolis, IN). Homogenates were clarified by sedimentation at 16,100×g for 10 minutes and supernatant immunoprecipitated using Dynal magnetic beads (Invitrogen, Carlsbad, CA) decorated with monoclonal antibodies against synaptophysin, or polyclonal antibodies against either myc or HA epitopes. After six washes with Buffer A 0.1% Triton X-100 for 5 min each, samples were loaded on 4–20% PAGE-SDS Criterion pre-cast gels (Bio-Rad, Carlsbad, CA) and analyzed by immunoblot. 1% to 2% of the initial homogenate was loaded as input.

### Sucrose sedimentation

Triton-X100 soluble supernatants from cells either treated with the crosslinker DSP or DMSO alone were separated in a 5% to 20% sucrose gradient prepared in buffer A plus 0.5% Triton X-100 during 13 hr at 187,000×g in a SW55 rotor [38]. Samples were collected from the bottom (250 µl/each) and analyzed by western blots. Gel filtration molecular weight markers (Sigma, saint Louis, MI) were used to calibrate the gradients: horse spleen apoferritin (443 kDa, 16.5 S), bovine serum albumin (66 kDa, 4.6 S), sweet potato β-amylase (200 kDa, 9.4 S) and bovine erythrocytes carbonic anhydrase (29 kDa, 2.9 S).

### Cell fractionation

PC12 cells differential fractionation and glycerol sedimentation were performed in intracellular buffer (38 mM potassium aspartate, 38 mM potassium glutamate, 38 mM potassium gluconate, 20 mM MOPS-KOH, pH 7.2, 5 mM reduced glutathione, 5 mM sodium carbonate, 2.5 mM magnesium sulfate, 2 mM EGTA) [36], [39]. Briefly, homogenate were sedimented 5 min at 1000×g to obtain a nuclear pellet (P1) and S1 supernatant that was sedimented at 27,000×g for 35 min to generate an S2 supernatant. S2 was loaded on the top of a 5% to 25% glycerol gradient. After 75 min at 218,000×g, samples were collected from the bottom. All gradient fractions were analyzed by immunoblot and immunoreactivity revealed by ECL. Immunoreactive bands were quantified using NIH Image 1.62 software as described [36].

### Zinquin staining and flow cytometry

PC12 cells treated for 30 min with 25 µM ZnSO4 were washed and incubated with 25 µM zinquin during 1 hr at 37°C. Cells were washed twice at 4°C and resuspended in PBS. Fluorescence was determined using a MoFlo High-performance Cell Sorter from Dako Cytomation (Fort Collins, CO) as described previously [36], [40] or using a Synergy HT microplate reader (BioTek Instruments Inc. Vermont) with an excitation∶emission filters of 340/30∶460/40.

### Molecular modeling

An initial molecular model for ZnT3 transporter was generated by the automated model server panther (http://bmcc3.cs.gsu.edu). The homologous zinc transporter Yiip [33] (pdb id code 2QFI) was found by profile-profile alignment. Even though the alignment has only a 14% identity, the close functional similarity and the significant profile alignment score strongly suggested that there was a true homology. Critically, the residues involved with zinc binding are preserved between the two molecules in this alignment. The initial model was built using the AMMP program [41], [42] with the latest potential set (atoms.tuna). The ZnT3 protein dimer was built by applying the non-crystallographic symmetry to the model that relates to the two monomers of Yiip in the 2QFI crystal structure. The zinc atoms from the 2QFI structure were used in the modeling, and the protein dimer model was further energy minimized after being generated. The tyrosine dimer was implemented using the preAMMP system and parameterized with the semi-empirical charge generation in AMMP [43]. The pairs 357,372' and 357', 372 where inserted into the coordinates and the model generated via energy minimization of the non-tyrosine bonded protein dimer model. As in all AMMP calculations, the amortized fast multipole method [44] was used to avoid artifacts due to non-bonded and electrostatic cutoffs. The electrostatic fields were calculated with full exponential Debye-Huckel expansion of the ionization potential using the finite element solver in AMMP. Models were made for the other possible pairs of tyrosine dimers, but these did not alter the zinc binding sites. Images of the molecular models where generated with Pymol 0.99 [45].

### Cell viability

PC12 cells expressing wt ZnT3 or ZnT3Y357F and ZnT3Y372F mutants were incubated during 24 hr with 200 µM to 250 µM ZnSO4. Cell viability was determined by trypan-blue exclusion using a Neubauer chamber. 100% viability was determined in the absence of zinc.

### Data Presentation

All data are depicted as average±standard error of the mean

## Results

### Stabilization of the oligomeric states of ZnT3 by cross-linking

The similarity of sequence between the *Escherichia coli* zinc transporter Yiip and mammalian zinc transporters of the SCL30A family led us to hypothesize that dimer formation could be a common structural element, shared by SLC30A mammalian zinc transporters. In this study, we focused on the human zinc transporter 3 (ZnT3) as a model of transmembrane protein representative of the SLC30A family. We took advantage of PC12 cells because they express low levels of ZnT3, are easily transfected with tagged versions of this transporter, and ZnT3 endosome/synaptic-like microvesicles (SLMVs) subcellular localization is well characterized in these cells [26].

**Figure 1:**
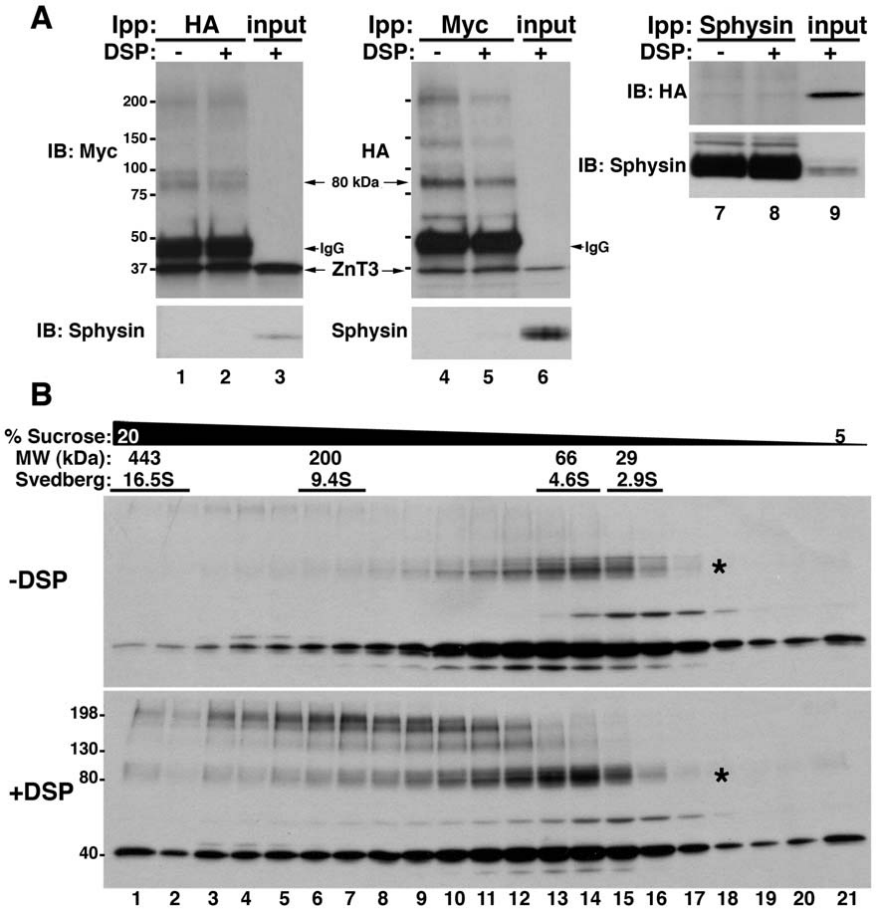
Identification of ZnT3 oligomeric states. A) Triton soluble extracts of PC12 cells (450 µg) co-expressing ZnT3-HA and ZnT3-myc, treated with and without cross-linker (DSP), were immunoprecipitated with HA (lanes 1 and 2), myc (lanes 4 and 5) or synaptophysin (Sphysin, lanes 7 and 8) antibodies. Western blots were probed with myc, HA and synaptophysin and ZnT3 antibodies, respectively. Control immunoprecipitation with synaptophysin antibodies fail to isolate ZnT3. Input 10 µg. B) 1.5 mg of Triton-X100 soluble supernatant of ZnT3-myc expressing cells treated in the presence of vehicle (DMSO) or DSP (+DSP) were separated by sucrose sedimentation. Fractions were collected from the bottom and analyzed by immunoblot with myc antibodies. An 80 kDa and high molecular weight forms of ZnT3 were observed, together with the monomeric 40 kDa species.

We examined the formation of ZnT3 oligomeric species by co-immunoprecipitation of ZnT3 carrying different tags in their carboxy terminal domains. In order to facilitate detection of low affinity ZnT3 oligomers (dimers and/or high molecular weight species) we used dithiobis–succinimidylpropionate (DSP). DSP is a homobifunctional cell permeable cross-linker. This agent contains a disulfide bond, which allows complete cleavage of cross-linked products under reducing conditions [38]. PC12 cells co-expressing ZnT3-HA and ZnT3-myc, treated with and without DSP, were lysed and detergent soluble supernatants immunoprecipitated with magnetic beads coated with HA or myc antibodies (Fig. 1A). Immune complexes were analyzed by immunoblot with antibodies against myc or HA.

Immunoprecipitation with antibodies against HA isolated myc-tagged ZnT3 (Fig 1A, left panel). Conversely, immunoprecipitation with myc antibodies recovered the HA-tagged transporter (Fig. 1A middle panel). Interaction between the tagged ZnT3s was observed either in the absence (Fig. 1A, lanes 1 and 4) or presence (Fig. 1A, lanes 2 and 5) of cross-linker. Moreover, an 80 kDa band as well as high molecular weight bands containing both ZnT3-HA and ZnT3-myc were also observed. These bands are resistant to SDS and reducing agents consistent with covalently modified ZnT3. Synaptophysin (sphysin), an abundant polytopic synaptic vesicle protein in PC12 cells, was used as a control. No ZnT3 was observed in immunoprecipitations performed with synaptophysin antibodies (Fig 1A, right panel). Furthermore, synaptophysin was absent from HA and myc immunoprecipitations excluding spurious membrane protein binding to myc or HA-antibody decorated beads.

We next sought to investigate the molecular weight of ZnT3 oligomeric species by sedimentation on density gradients. Triton-X100 soluble supernatant of PC12 cells expressing ZnT3-myc, treated in the absence or presence of DSP, were separated by sucrose sedimentation [38], followed by reducing SDS-PAGE to revert cross-linking (Fig. 1B). The majority of the 40 kDa monomeric ZnT3 migrated at or above 4.6S, a sedimentation coefficient that exceeds the migration expected for a ∼40 kDa membrane protein (Fig. 1B) [38]. Moreover, the myc-tagged ZnT3 species of 80 kDa was also observed migrating at 4.6 S either in the absence or presence of cross-linker (see asterisk Figure 1 B, upper panel and lower panel). The 80 kDa band was resistant to reducing agents and SDS and increased in abundance in the presence of DSP. Moreover, new species of 160 and 240 kDa appeared sedimenting between 9.4 and 16.5S. These results show that ZnT3 forms covalent oligomeric structures, which are at least compatible with ZnT3 dimers and high molecular weight oligomers. Furthermore, a cell permeant cross-linking agent stabilizes these oligomeric species.

### ZnT3 80 kDa species is increased by oxidative stress

**Figure 2:**
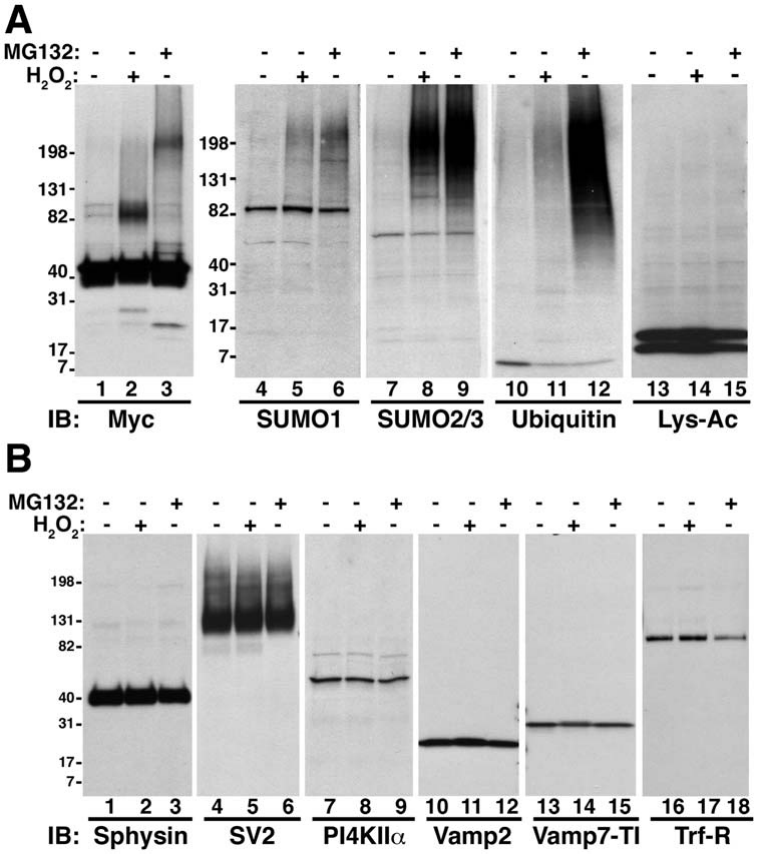
Covalently modified species of SLC30A family members are induced by oxidative stress. PC12 cells incubated without or with 100 mM H_2_O_2_ for 20 min or 10 µM MG132 for 12 hours were analyzed by immunoblot with antibodies against A) myc, SUMO1, SUMO2/3, ubiquitin or acetylated lysine. Human ZnT3 putative dimeric form (80 kDa band) and high molecular weight species were observed in H_2_O_2_ and MG-132 incubations, respectively. B) Effect of H_2_O_2_ and MG132 treatment in components of different intracellular compartments were analyzed by immunoblot with transferring receptor (Tfr-R, early/recycling endosomes), Vamp7-TI (late endosomes) and synaptophysin (Sphysin), SV2 and PI4KIIα (SLMVs) antibodies.

The reduced SDS-PAGE migration of ZnT3 suggested that this transporter could form dimeric species. Alternatively, increased ZnT3 molecular weight might also occur by covalent post-translational modifications, such as sumoylation, multi mono-ubiquitylation and poly-ubiquitylation. To distinguish between these two hypotheses, H_2_O_2_ and the inhibitor of the proteasome MG-132, were used to increase general sumoylation [46] and ubiquitylation [47], respectively (Fig. 2A). Incubation with H_2_O_2_ increased the abundance of the human ZnT3 80 kDa form (Fig. 2A, lane 2), compared with control. In contrast, treatment with MG-132 (Fig. 2A, lane 3) induced the accumulation of high molecular weight ZnT3-myc complexes (>200 kDa). These complexes are probably formed in response to oxidative stress induce by MG-132, since they were not detected with antibodies against ubiquitin (data not shown). As expected, both treatments increased the abundance of sumoylated and ubiquitylated proteins, verified by immunoblot with antibodies against SUMO1, SUMO2/3 and ubiquitin (Fig 2A, lanes 4–12). Lysine acetylation, another post-translational modification, was unaffected in both conditions (Fig 2A, lanes 13–15). H_2_O_2_ or MG-132 treatments showed no effect on other synaptic vesicle membrane proteins, such as synaptophysin, synaptobrevin 2 (Vamp2), SV2 or phosphatidylinositol-4-kinase type II alpha (PI4KIIα), the late endosome/lysosome SNARE Vamp7 and PI4KIIα, or the endosomal marker transferrin receptor (Tfr-R) (Fig. 2B). These observations indicate that the increase in molecular weight of ZnT3, is not the result of unspecific aggregation of membrane proteins where ZnT3 resides.

### The ZnT3 80 kDa species is a covalent dimer

SUMO1 antibodies detected a band of 80 kDa in PC12 cells harboring myc tagged human ZnT3 (Fig 2A, lanes 4–6). Thus, we hypothesized that the increase in molecular weight induced by H_2_O_2_ was due to ZnT3 sumoylation. To test this hypothesis, we transfect HA-tag versions of SUMO1, SUMO2 or SUMO3 into PC12 cells expressing human ZnT3-myc. Protein sumoylation was induced by incubating cells in the presence of H_2_O_2_ or MG132 (Fig. S1). Immunoprecipitation of SUMO 1, 2 or 3 using HA antibodies isolated a wide range of sumoylated proteins and free SUMO, from control cells, but failed to isolate ZnT3-myc immunoreactive bands (Fig. S1A). Moreover, induction of sumoylation by H_2_O_2_ incubations (Fig. S1B), although it increased the amount of the 80 kDa ZnT3-myc species observed in the inputs (Fig. S1B, lanes 14–16), did not lead to ZnT3-myc immunoreactive material in SUMO-HA immunoprecipitations (Fig. S1B, lanes 4–12). No ZnT3 species were observed in HA immunoprecipitations of MG-132 treated cells expressing SUMO constructs (Fig. S1B, compare lanes 6, 9 and 12 with lane 3). Moreover, immunoprecipitations with myc antibodies and western blots with ubiquitin antibodies did not identify any ubiquitinated products (data not shown). These results exclude that the ZnT3 80 kDa or oligomeric species are sumoylated or ubiquitinated products and support the hypothesis that the 80 kDa species is a covalent ZnT3 dimer. Moreover, our findings indicate that the ZnT3 80 kDa covalent species is increased by oxidative stress.

### ZnT3 covalent dimer formation depends on tyrosines in its carboxy terminal domain

**Figure 3:**
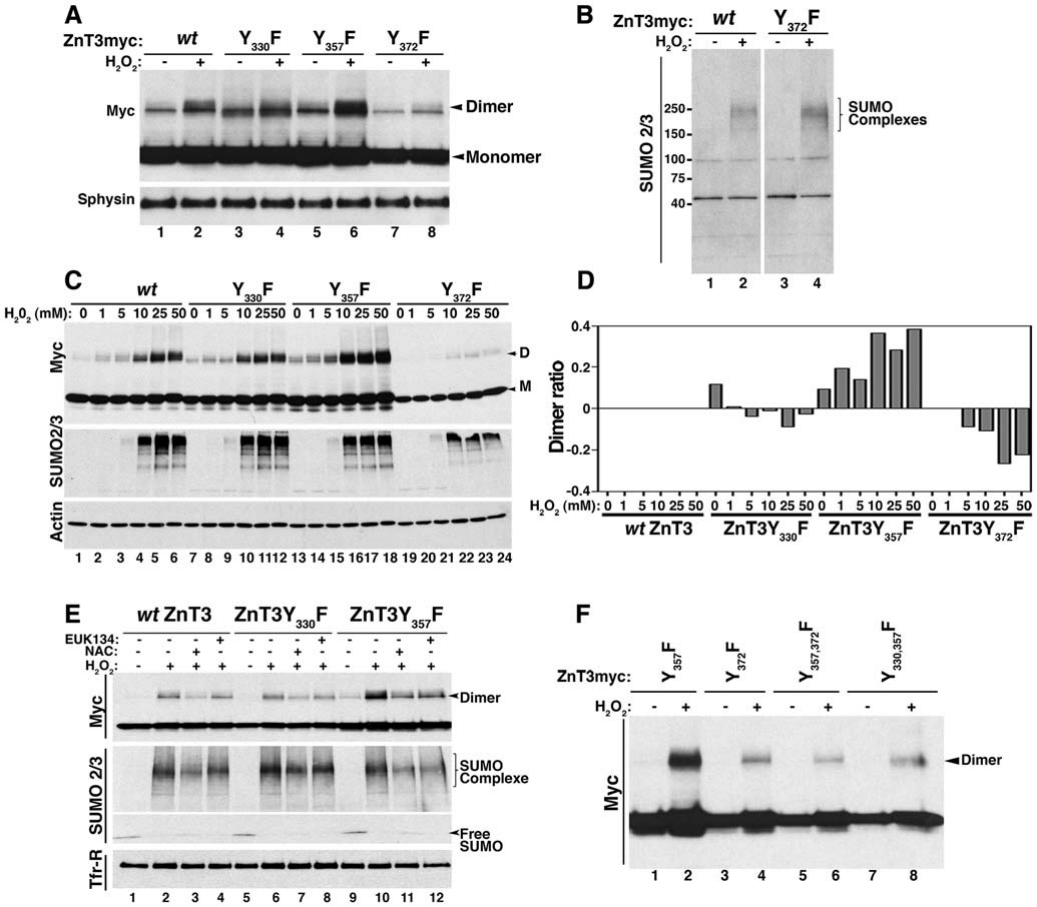
Covalent ZnT3 oligomeric species require tyrosine residues and free radicals for their formation. Transiently transfected (A and B) or permanent (C) PC12 cell lines expressing human ZnT3-myc *wild type* or mutants in tyrosine 330, 357 or 372 were incubated without or with 10 mM (A and B) or increasing concentrations (C) of H_2_O_2_ during 1 hr in RPMI medium. 20 µg of total extracts were analyzed by immunoblot with the indicated antibodies. D and M denotes dimer and M monomer, respectively. D) Dimer formation was expressed as a ratio of dimer versus monomer for each H_2_O_2_ concentration as follow: (mutant ratio of dimer/monomer)- (wild type ratio of dimer/monomer). In the case of *wild type* ZnT3 the expected value is 0. Increase in ZnT3 dimer content is seen as positive values whereas a decrease corresponds to negative values. E) PC12 cells expressing ZnT3-myc *wild type* or tyrosine mutants ZnT3Y357F and ZnT3Y375F were pre-treated during 15 min without or with 10 mM N-acetyl cysteine (NAC) or 50 µM EUK138 before incubation with 10 mM H_2_O_2_ during 1 hr. F) PC12 cells expressing single and double mutants were treated with or without 10 mM H_2_O_2_ lysed and analyzed by western blot with myc antibodies.

One modification that could explain formation of covalent dimers regulated by oxidative stress is dityrosine bond formation [48]. Based on the chemistry of dityrosine synthesis, we selected two criteria to identify dityrosine bonds in the 80 kDa ZnT3 species [48]. First, dityrosine formation is induced by oxidative stress and second, is abrogated by tyrosine to phenylalanine mutation of critical residues. We previously showed a ZnT3 80 kDa band increased by oxidative stress, thus meeting the first requirement. We next tested whether tyrosine to phenylalanine mutations in ZnT3 tyrosine residues could abrogate the H_2_O_2_-induced dimer formation. Human ZnT3 possesses six tyrosine residues, two facing extracellular exposed domains, one in an intracellular loop, and three in the carboxy-terminal domain. We focused on the role of individual tyrosine residues present in the carboxy-terminal domain as candidates to mediate dimer formation (Y330, Y357 and Y372). Tyrosine to phenylalanine mutations were engineered in human ZnT3-myc and transiently transfected in PC12 cells, which were incubated with H_2_O_2_ (Fig. 3A). Mutation of tyrosine 372 shows a reduced amount of dimers compared with control (Fig. 3A, compare lanes 2 and 8). In contrast, Y357F increased dimerization (Fig. 3A, compare lanes 2 and 6). No differences in the response to oxidative stress, detected with SUMO2/3 antibodies, was observed between cell expressing Y372F mutant or *wt* ZnT3 (Fig. 3B). To further evaluate these differences permanent cells lines carrying *wild type* and mutant human ZnT3-myc were incubated with increasing concentrations of H_2_O_2_ (Fig. 3C). ZnT3 80 kDa species were quantified for each H_2_O_2_ concentration and expressed as a ratio of ZnT3 80 kDa species versus monomer. To determine differences with respect to *wild type* ZnT3, ratios obtained from *wild type* and tyrosine mutants were subtracted from the *wild type* human ZnT3 ratio (Fig. 3D, dimer ratio = 0 for *wild type* ZnT3). Much like in transiently transfected cells, two phenotypes were observed in cell lines expressing human ZnT3 mutant transporters. First, mutation of Y372F drastically reduced dimerization under control conditions (Fig. 3C, compare lane 19 with lane 1), as well as in response to oxidative stress (Fig. 3C lanes 20 to 24) when compared to *wild type* ZnT3 (Fig. 3C, lanes 2 to 6). In contrast, Y357F mutation increased dimerization both in the absence (Fig. 3C, compare lane 13 with lane 1) or presence of H_2_O_2_ (Fig. 3C, compare lanes 14 to 18 with lanes 2 to 6). The ZnT3 Y357F mutant increased ZnT3 80 kDa species 2 to 4 fold when compared to *wild type* after H_2_O_2_ incubation. No significant differences were observed in cells expressing the Y330F mutant irrespective of whether this mutant was expressed transiently (Fig. 3A, compare lanes 2 and 4) or in a stable PC12 cell line (Fig 3C, compare lanes 7–12 with 2–6). Immunoblot with SUMO2/3 antibodies showed that all cell lines were responsive to H_2_O_2_-induced oxidative stress.

To further assess the role of oxidative stress on human ZnT3 tyrosine-dependent dimerization, we asked whether the formation of the ZnT3 80 kDa species could be prevented by pre-incubation with anti-oxidants. We compared *wild type* human ZnT3 and the Y330F and Y357F mutants. These mutants either do not affect the formation of the ZnT3 80 kDa species or increased its formation, respectively. The effects of H_2_O_2_ on dimer formation were completely abolished by catalase (data not shown) and significantly reduced by incubations with the free radical scavengers N-acetyl-cysteine (NAC, Fig. 3E lanes 3, 7 and 11) and EUK138 (Fig. 3E. lanes 4, 8 and 12) in all three cell lines. All together, these results demonstrate that the 80 kDa form of ZnT3 is a tyrosine dimer formed by a covalent bridge mediated mainly by tyrosine 372 in the carboxy terminal domain.

We further explored whether tyrosine residues in the cytosolic tail of human ZnT3 exhibited a dimerization hierarchy in response to oxidative stress. We specifically asked whether human ZnT3 harboring the Y372F mutation, which reduces the ZnT3 80 kDa species, was dominant over the Y357F mutation, which increases ZnT3 80 kDa species. Analysis of double mutants ZnT3Y357-372F showed reduced ZnT3 80 kDa species indicating that Y372 is critical and dominant in the formation of dityrosine dimers (Fig 3F, lanes 5–6). Moreover, these results indicate that the remaining Y330 present in ZnT3Y357-372F were less efficiently engaged in dimer formation with other Y330 residue. We next asked whether tyrosine dimers were formed between two Y372 residues. ZnT3 carrying only one tyrosine residue in position 372 by mutation of Y330–357 to F still displayed reduced ZnT3 80 kDa species content (Fig 3F, lanes 7–8). Thus, tyrosine dimers are not formed between two adjacent Y372 residues. These results argue for a model where the ZnT3 80 kDa predominant species is formed in trans between residues 372 and 357.

### Formation of ZnT3 tyrosine dimers regulates ZnT3 targeting to synaptic-like microvesicles (SLMV)

**Figure 4:**
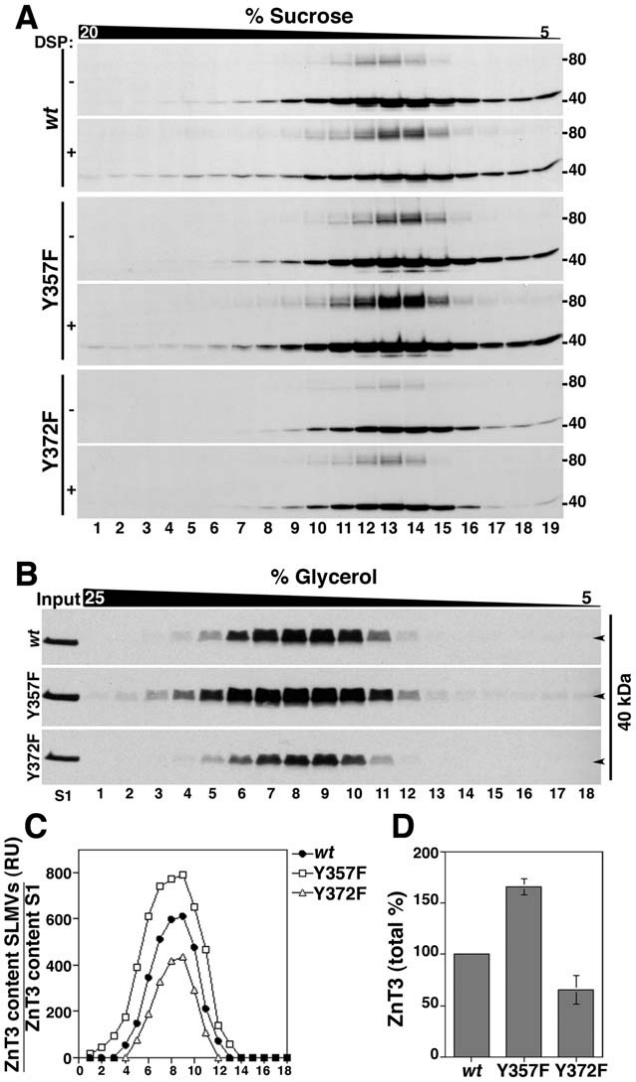
Tyrosine-dependent covalent oligomerization regulates ZnT3 targeting to PC12 SLMVs. A) Triton soluble extracts of PC12 cells treated with and without cross-linker, were separated in a 5–20% sucrose gradient. 19 fractions were collected from the bottom (fraction 1) and analyzed by immunoblot with myc antibodies. Either reduced or increased levels of dimerization were observed in ZnT3Y372F and ZnT3Y357F mutants in the presence DSP, respectively. B) PC12 stable cell lines expressing *wild type* ZnT3 and ZnT3Y357F and ZnT3Y372F mutants were fractionated as described in Methods. S2 (600 µg) supernatants were separated in glycerol gradients and ZnT3 distribution analyzed by immunoblot with myc antibodies. SLMVs peak, in the middle of the gradient. ZnT3 was quantified (C) and adjusted by expression levels in 10 µg of S1 (B, input). D) Total levels of ZnT3 tyrosine mutants in the gradients were compared with wild type ZnT3 (100%) in three independent experiments. ZnT3Y357F mutant: 165.3%±7.7%. ZnT3Y372F mutant: 65.5±14%.

Dityrosine formation has been described mainly as a product of oxidative stress [48] and as a normal extracellular post-translational modification only in a limited group of secreted proteins, such as collagen and elastin [11], [13]. We used cross-linking and sucrose sedimentation to evaluate whether dimer formation in tyrosine mutants occurs spontaneously in the absence of H_2_O_2_ as we observed previously for *wild type* ZnT3 (Fig. 1B). Triton-X100 soluble extracts of PC12 expressing *wild type* human ZnT3-myc, ZnT3Y357F and ZnT3Y372F mutants treated with or without DSP were separated by sucrose sedimentation and western blots analyzed with myc antibodies (Fig. 4A). As we observed before, the ZnT3Y357F mutant showed a strong increase of dimeric forms even in the absence of DSP and the content of the ZnT3 80 kDa form was further increased by DSP treatment. On the contrary, Y372F mutant present a reduced dimerization that was modestly increased by the addition of DSP (1.3 folds compared with 2.2 folds for *wt* ZnT3). These results indicate that dimers are formed spontaneously and that DSP stabilizes ZnT3 oligomeric transient intermediaries.

We hypothesized that if dimerization occurs spontaneously it could regulate human ZnT3 function either by affecting its subcellular localization and/or zinc transport activity. To test this hypothesis, we took advantage of ZnT3 mutants that either decrease or enhance the formation of the 80 kDa species. ZnT3 localized mainly in synaptic-like microvesicles (SLMV) and endosomes in PC12 cells [26], [49]. To investigate whether tyrosine dimerization affects ZnT3 vesicle targeting at steady state, S2 fractions containing SLMVs were resolved by glycerol velocity sedimentation from cell lines expressing *wild type* human ZnT3 and tyrosine mutants (Fig. 4B). SLMVs sediment in glycerol based on organelle size and they characteristically peak at the middle of glycerol gradients [50], [39] . In these gradients, changes in the targeting of membrane proteins to SLMVs is frequently seen as modification in the amount but not sedimentation of a marker [51]–[53].

ZnT3 targeting to SLMVs was determined in vesicles resolved in glycerol gradients. Results were quantified and normalized by the expression level in the initial fractionation input (Fig. 4C and D). Human ZnT3 carrying the Y357F mutation, which possesses an increased formation of ZnT3 80 kDa species, increased its targeting to SLMVs by 65%±7.76 (n = 3). In contrast, human ZnT3 harboring the Y372F mutation, which reduces the formation of the 80 kDa species, decreased its targeting by 35%±14.3% (n = 3) with respect to *wild type* human ZnT3 (Fig. 4D). These results indicate that tyrosine-mediated dimer formation regulates human ZnT3 targeting to SLMVs.

### Tyrosine dimerization regulates zinc transport capacity

ZnT3 increases vesicular zinc content when present in SLMVs [40]. Thus, we explored whether the changes in ZnT3 SLMV targeting due to a Y to F mutation correlated with modifications in zinc uptake. Organelle zinc stores were detected using zinquin, a zinc specific fluoroprobe [54]. As previously reported [23], [36], [40], vesicular zinc level was minimally affected by the over-expression of the mouse ZnT3 ortholog when compared with non-transfected cells (5A, mouse ZnT3, 1.1±0.04). In contrast, expression of the human ZnT3 increased zinc levels ∼2.5 fold (2.41±0.51, p<0.001). A significant increase in vesicular zinc stores was detected in cells expressing human versions of the intracellular zinc transporter ZnT4 (1.69±0.09, p = <0.001) and ZnT5 (2.27±0.2, p<0.001), but not ZnT1, a zinc transporter localized in the plasma membrane, that mediate zinc efflux to the extracellular milieu (1.12±0.26, p = 0.21). Human ZnT7 expression shows a modest 24% increase with respect to non-transfected cells (1.24±0.04, p<0.001).

**Figure 5:**
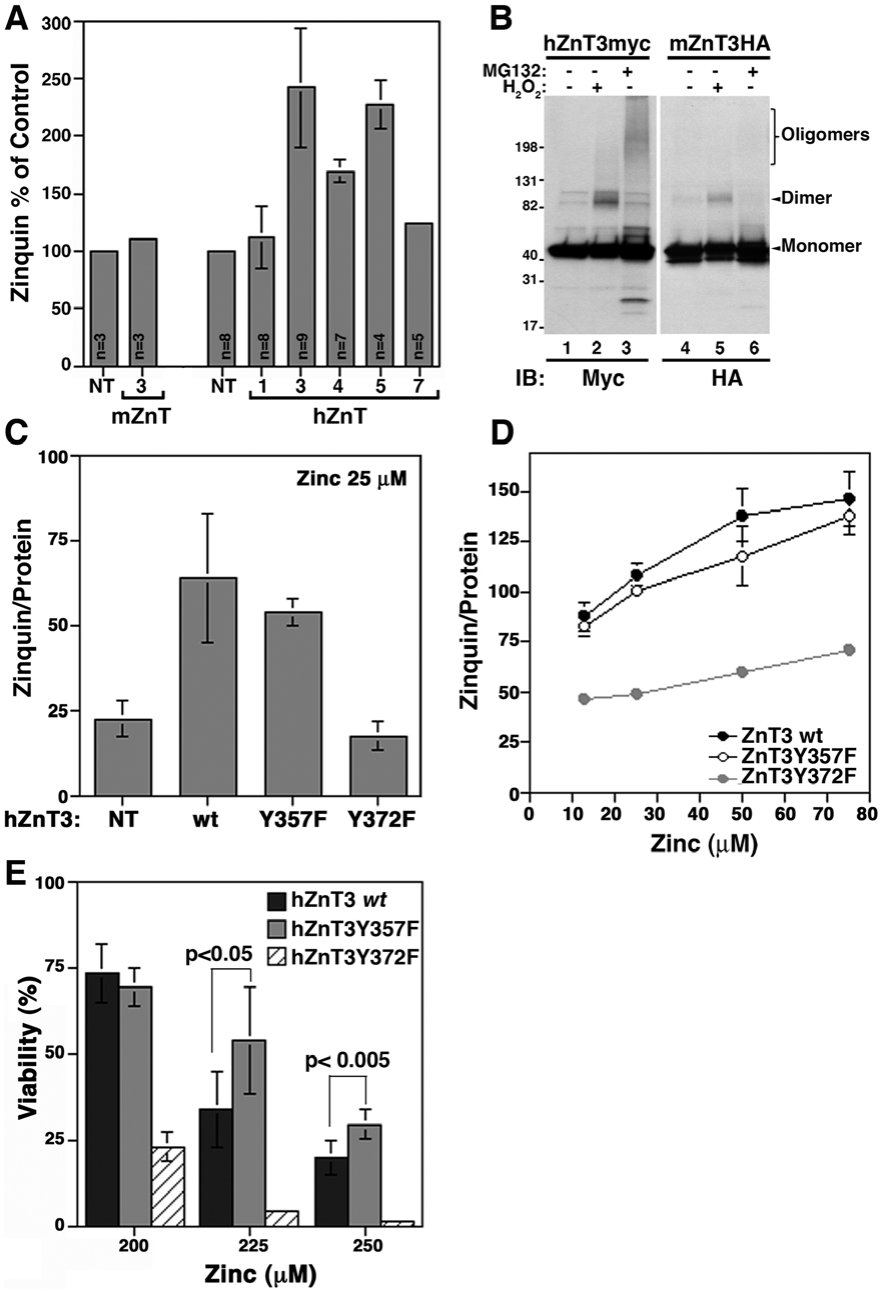
Vesicular zinc storage is modified by mutants that affect tyrosine-mediated ZnT3 oligomer formation. Vesicular zinc storage was measured with zinquin in permanent cells lines as described in Methods. A) PC12 cells non transfected (NT) and cells expressing mouse ZnT3HA or myc-tag human ZnT1, 3, 4, 5 and 7 were stained with zinquin and fluorescence analyzed by flow cytometry, results are express as mean±SD in three independent experiments for human ZnTs. hZnT1: 112±26.5, hZnT3: 241±51.3, hZnT4: 169±9.7, hZnT5 227±20.3, hZnT7: 124±4.19. For mouse ZnT3-HA results are mean±SD in one experiment by triplicate. B) Cell lines expressing mouse ZnT3-HA or human ZnT3-myc were incubated with or without H_2_O_2_ or MG132 and extract analyzed by immunoblot with myc and HA antibodies. C and D) Vesicular zinc storage was measured in a microplate reader. Non-transfected cells (NT) and cells expressing myc-tag human ZnT3 (*wild type*), ZnT3Y357F or ZnT3Y372F mutants were incubated with the indicated concentration of ZnSO_4_. Zinquin staining was expressed by µg of protein. NT: 22.4±5.4, *wild type* ZnT3: 63.8±18.8, Y357F: 53.7±3.8. Y372F: 17.4±4.3, n = 3 independent experiments. E) Permanent PC12 cell lines expressing *wt* ZnT3 and ZnT3Y357F or ZnT3Y372F mutants were incubated with the indicated concentration of ZnSO_4_ for 24 hr. Cell viability was measured by trypan-blue exclusion as described in Materials and Methods. Y357F mutant increased viability from 34±11.3 (*wt*) to 54.3±15 (p<0.05) at 225 µM and from 20.1 (*wt*) ±5 to 29.9±4.3 (p<0.005) at 250 µM (n = 5 in two independent experiments). In contrast, loss-of-function Y372F mutant decreases cell viability to 4.5±0.48 (p<0.001) and 1.57±0.71 (p<0.0001), respectively (n = 6 in two independent experiments.

The differences in zinc uptake by human and mouse ZnT3 orthologues prompted us to evaluate the capacity of mouse ZnT3 to form oligomers. PC12 cells expressing human or mouse ZnT3 were incubated with H_2_O_2_ or MG132, and the formation of oligomeric species compared by western blots under reducing conditions (Fig. 5B). Notably, a small proportion of the 80 kDa species was observed with mouse ZnT3-HA in the presence of H_2_O_2_ when compared with the human transporter (Fig. 5B, compare lane 5 with 2). Moreover, mouse ZnT3 seems to be insensitive to oligomerization induced by MG132 (Fig. 5B, compare lane 6 with 3). Based on these observations, we hypothesized that zinc transport is mediated by ZnT3 oligomeric species. A prediction that arises from this hypothesis is that the prevention of dimerization by tyrosine mutation should prevent zinc transport in the human ZnT3. Non-transfected PC12 cells (NT) and cells transfected with either *wild type* human ZnT3-myc or ZnT3 tyrosine mutants were stained with zinquin (Fig. 5C). Similar levels of vesicular zinc accumulation were observed in both *wild type* human ZnT3 (63.8±18.8) and the Y357F mutant capable of increased oligomer formation (53.7±3.8). On the contrary, the Y372F mutant that prevents human ZnT3 oligomer formation showed basal zinquin staining similar to non-transfected cells (Fig. 5C). Incubation with increasing concentrations of ZnSO_4_ further demonstrated the inability of the Y372F mutant to support zinc accumulation into vesicular compartments (Fig. 5D). These results are consistent with a model whereby human ZnT3 oligomers stabilized by dityrosine bonds are required for efficient zinc transport.

We hypothesized that the inability of the Y372F mutant to support zinc storage in vesicular compartments would affect cell viability when cells are challenged with toxic extracellular zinc concentrations. To test this hypothesis, PC12 cells lines expressing *wild type* human ZnT3 and tyrosine mutants were cultured in media containing increasing concentrations of ZnSO_4_ during 24 h (Fig. 5E). Cells expressing human ZnT3 carrying the Y372F mutation, which impairs ZnT3 dimerization, were sensitive to extracellular zinc. Cell viability was reduced when compared with cells expressing *wild type* human ZnT3. In contrast, cells expressing human ZnT3 carrying the Y357F mutant, which enhances ZnT3 dimerization, showed a modest but significant increase in cell viability compared with human *wild type* ZnT3 (Fig. 5E). These findings indicate that, although modest, a gain-of-function phenotype in human ZnT3 carrying the Y357F mutation becomes evident in cell challenged for a prolonged time with toxic zinc concentrations.

### Predicted structural changes induced by ZnT3 dityrosine bond oligomerization

**Figure 6:**
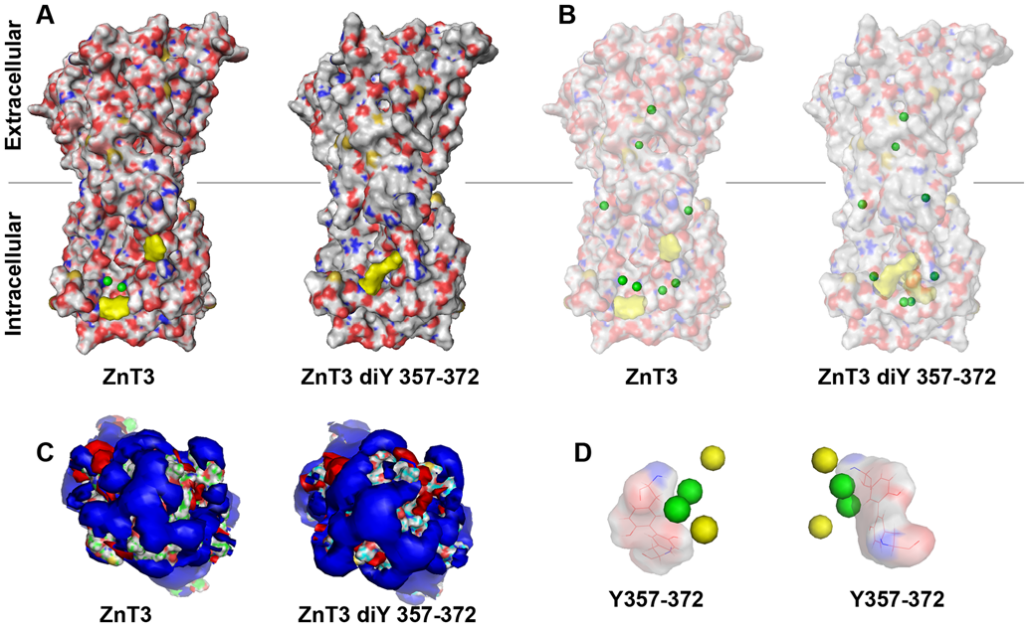
ZnT3 homology modeling with the bacteria Yiip zinc transporter. Human ZnT3 pairs or ZnT3 pairs bridged in trans by dityrosine bonds between tyrosine residues 357 and 372 were modeled using AMMP and visualized with Pymol. Modeling coordinates were obtained from the crystal structure of YiiP bound to zinc atoms. A) Depicts lateral views of ZnT3 pair surface models. Gray lines represent the middle of the lipid bilayer. Zinc atoms are depicted as green spheres. Tyrosines 357 and 372 are depicted either single or bonded in yellow. B) Depicts diaphanized surface models to highlight the position of zinc atoms (green spheres). C) Cytoplasmic views of the solvent exposed areas in the absence or presence of dityrosine bonding. Blue depicts the negative and red the positive potentials. D) Cytoplasmic view of zinc atoms arrangement in the cytosolic domain of ZnT3 pairs either in the absence of dityrosine bonds (green spheres) or in the presence of dityrosines (yellow spheres). Dityrosines bonds are represented as a reference point.

Dityrosine bonded ZnT3 supports efficient zinc accumulation in intracellular organelles. This functional change predicts that ZnT3 oligomers containing trans dityrosine bridges between residues 357 and 372 should modify cytoplasmic determinants involved in zinc binding. We explored the structural changes induced by bridging tyrosines 357 and 372 in ZnT3 dimers using AMMP molecular modeling. We modeled human ZnT3 primary sequence (Fig. 6) using as a backbone the crystal structure coordinates of the bacterial ZnT3 homologue YiiP bound to zinc atoms [33]. ZnT3 dimers lacking dityrosine bonds closely resembled the crystal structure of YiiP (Fig. 6A, ZnT3). Zinc atoms in human ZnT3 bound to the cytosolic domain were exposed to water. However, ZnT3 dimers carrying a dityrosine bond between residues 357 and 372 acquired a closed conformation with zinc atoms bound to the cytosolic domain completely buried and away from solvent (Fig. 6A, ZnT3 diY357–372). The major conformational change involved in the 357–372 dityrosine dimer depended on the motion of the two cytoplasmic loops containing these two tyrosines. Since tyrosine 372 is on the C-terminus and therefore has few conformational restrictions, it moves more than tyrosine 357 (Fig. 6A). The modeling method was unlikely to produce major conformation changes as it used conjugate gradients, which is a local optimizer. The distribution of charges, as reflected in the electrostatic field calculated from the model in the presence of dielectric and counter ions showed few differences between the undimerized and dimerized tyrosine residues (Fig. 6C). The arrangement of zinc atoms was further explored by projecting cytosolic zinc atoms into the dityrosine bonds (Fig. 6D). Zinc atoms moved closer to the unexposed surface of the C-terminal domain of ZnT3 in dimers carrying 357–372 dityrosine bonds (Fig. 6D, compare yellow and green dots). Models made for other pairs of possible dityrosine states, such as 330–357 or 330–372 did not alter the zinc binding sites. The rearrangement seen with the 357–372 dityrosine bridge appears to alter the zinc binding sites and open up buried binding sites that are not present in molecules lacking dityrosine bonds (Fig. 6B). Zinc atoms not associated with this binding site are buried in both the tyrosine and dityrosine models. After formation of the dityrosine bond a complete set of well-formed zinc binding sites is generated. This set of sites spans the whole length of the molecule suggesting that dityrosine formation facilitates zinc transport by forming the shielded binding pathway for the C-terminal part of the transporter. This modeling supports the notion that ZnT3 domains involved in zinc binding undergo structural rearrangements in the presence of dityrosine bonds.

### Structural determinants defining dityrosine-dependent dimerization in SLC30A family members

**Figure 7:**
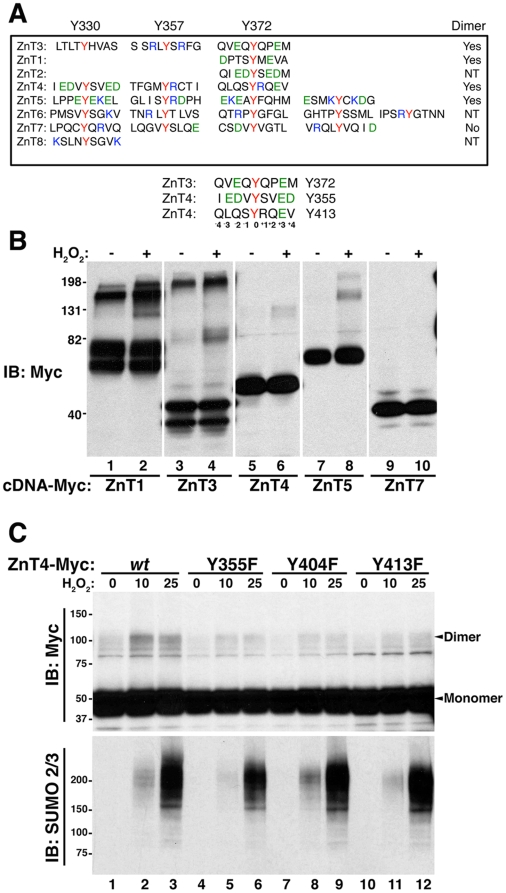
Dityrosine-dependent dimerization in SLC30A family members. A) Carboxy terminal primary sequences of ZnTs 1–8 containing tyrosine residues are compared with ZnT3 tyrosines 330, 357 and 372. Negatively (E/D) and positively (R/K) charge residues are depicted in green and blue, respectively. ZnT3Y372 and ZnT4Y355 and 413 share a conserved YXXE sequence. NT denotes not tested. B) HEK293 cells were transiently transfected with plasmids encoding myc-tagged versions of human ZnT1, 3, 4, 5 and 7. Cell extracts were analyzed by immunoblot with myc antibodies. All zinc transporters tested, except ZnT7 showed covalently modified species resistant to SDS and reducing agents in the presence of H_2_O_2_. C) HEK293 cells transiently transfected with myc-tagged versions of *wild type* ZnT4 and Y355F, Y404F and Y413F mutants were incubated with the indicated concentrations of H_2_O_2_ during 30 min. Samples were analyzed by western blots with myc and SUMO2/3 antibodies. All three ZnT4 tyrosine mediated covalent tyrosine dimers.

ZnTs 1 to 8 possess C-terminal tyrosines that could mediate dityrosine bonding, as it is the case of human ZnT3 (Fig. 7A). To explore whether dityrosine bonds could be a common structural element in other SLC30A family members, we first examined whether myc-tagged versions of human ZnTs form covalent dimers in response to H_2_O_2_ (Fig. 7B). Similar to the human ZnT3, H_2_O_2_ induced covalently modified species in myc-tagged versions of human ZnT1, ZnT4 and ZnT5, but not ZnT7 (Fig. 7B).

Site directed mutagenesis and molecular modeling indicated that Y357-Y372 is the main bond involved in the dimerization of human ZnT3. Primary sequence analysis revealed that human ZnT3 residue Y372 is flanked by negatively charge amino acids (Fig. 7A). We searched whether other ZnT transporters possess Y residues surrounded by negatively charged residues. Human ZnT4 residues Y355 and Y413 are adjacent to a glutamic residue in position +3 from the tyrosine [YXXE] (Fig. 7A). In contrast, ZnT6, 7 and 8 contains tyrosines that lack negative residue in position +3. Interestingly, ZnT7 did not show H_2_O_2_-induced dimerization (Fig. 7B). To test whether at least one of the Y residues participating in dityrosine bonding needs to conform to the putative consensus YXXE, we performed site-directed mutagenesis of either the human ZnT4myc residue Y355 or Y413. *Wild type* and mutant ZnT4 constructs were expressed in HEK293 cells. Tyrosine to phenylalanine mutagenesis of either ZnT4 residues Y355 or Y413 decreased H_2_O_2_-induced dimerization compared with *wild type* ZnT4 (Fig. 7C). Similarly, mutagenesis of Y404F reduced dimer formation (Fig. 7C) indicating that ZnT4, like ZnT3, forms tyrosine dimers in which at least one of the Y residues abides to a putative YXXE consensus.

**Figure 8:**
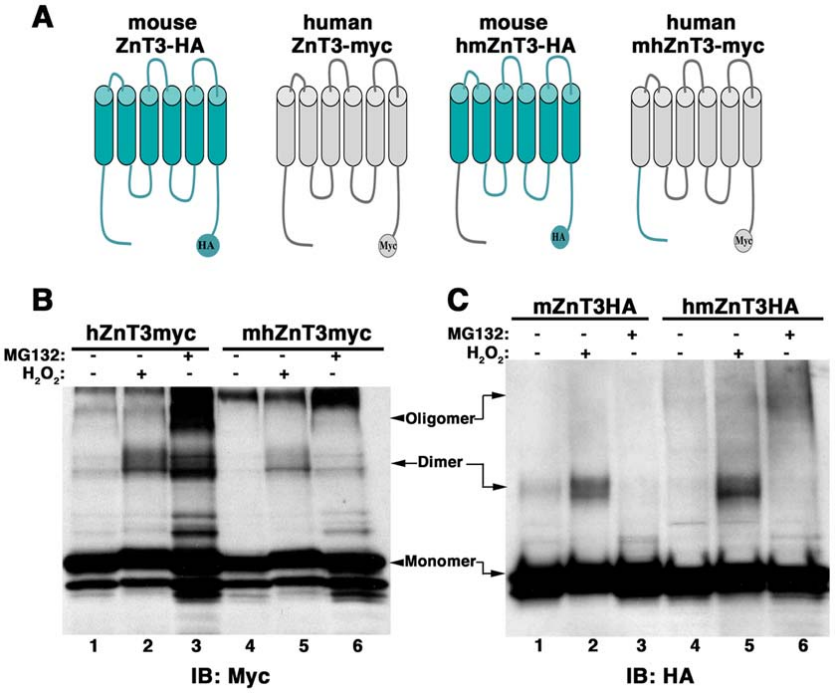
ZnT3 amino terminal domain regulates tyrosine dimerization. A) ZnT3 mouse and human chimeras, hmZnT3-HA and mhZnT3-myc, in which amino terminal domains (1–75) were exchanged were incubated with or without H_2_O_2_ or MG132 (B and C). Oligomerization was then compared with *wild type* mouse and human ZnT3. B) The amino terminal domain of mouse ZnT3 decreases human transporter's oligomerization. C) Human amino terminal increases mouse ZnT3 tyrosine oligomerization.

Mouse and human ZnT3 dimer formation differs dramatically despite the fact that both ZnT3 orthologs are 86% identical. This led us to hypothesize that additional structural elements could modulate the ability of tyrosine residues to form covalent bonds. To test this hypothesis, we asked whether the amino terminal domain of human ZnT3 could confer sensitivity to oxidative stress-induce oligomerization to the mouse ZnT3 and vice verse. To this end, we constructed a mouse ZnT3-HA chimeras containing the amino terminal of human ZnT3 (hmZnT3-HA) and a human ZnT3-myc chimera containing the amino terminal of mouse ZnT3 (mhZnT3-myc) (Fig. 8A). Constructs were transfected in PC12 cells and cells incubated with H_2_O_2_ or MG-132 (Fig. 8B and C). Mouse ZnT3 amino terminal decreased the H_2_O_2_-induced dimerization of the human chimera mhZnT3-myc (Fig. 8B). In contrast, the human amino terminal domain increases dimerization of the mouse ZnT3 chimera (hmZnT3-HA) in response to H_2_O_2_ and oligomerization-induced by MG-132. Therefore, the amino terminal domain influences the ability of carboxy-terminal tyrosines to form covalent tyrosine bonds.

## Discussion

**Figure 9:**
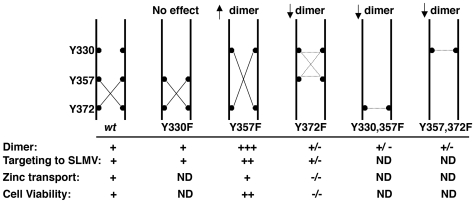
Model of ZnT3 dimer formation deduced from single and double tyrosine mutations. ZnT3 carboxy-terminal domains of two adjacent ZnT3 molecules are depicted by two vertical parallel black lines. Tyrosine residues and their position are illustrated in black circles. Lines connecting black circles depict covalent tyrosine bonds. Single and double mutant indicate that the predominant dimer is formed between Y372-Y357. Sorting to SLMVs and zinc transport was evaluated in the gain-of-function ZnT3Y357F and the loss-of-function ZnT3Y372F mutants. Increased dimerization of the ZnT3Y357F mutant increased its targeting to SLMVs without affecting its zinc transport capacity, measured by zinquin staining, but increase resistance to zinc toxicity. In contrast, mutation of Y372 decreases SLMV sorting, completely prevents zinc transport and increase cell toxicity to zinc. +/− denotes reduce respect to *wild type* and ND experiment not done.

In this study we demonstrate that tyrosine dimerization in a polytopic transmembrane protein, the zinc transporter 3 (SLC30A3/ZnT3), regulates the transporter's subcellular localization and its transport capacity. These functional changes correlate with structural rearrangements within the cytosolic domain of ZnT3 that favor the accessibility of zinc to metal binding sites, as suggested by molecular modeling studies. ZnT3 tyrosine dimer formation occurs spontaneously and it is induced by oxidative stress. Our conclusions are supported by phenotypes resulting from discrete tyrosine to phenylalanine mutants, illustrated in Fig. 9. Three tyrosines in the carboxy-terminal of ZnT3 were subject to site directed mutagenesis Y330 (TLTYHVA), Y357 (SRLYSRF) and Y372 (VEQYQPE). ZnT3 carrying the Y357 to phenylalanine mutation behaved as “gain-of-function” mutant, with increased dimer formation, enhanced ZnT3 targeting to SLMVs and cell protection to zinc toxicity. In contrast, mutation of Y372 induced a “loss-of-function” phenotype, with reduced dimerization, decrease SLMV targeting, impaired zinc transport into vesicle compartments and decrease protection to zinc toxicity.

Until now, dimerization of polytopic transmembrane proteins has been shown to occur by covalent and non-covalent interaction mainly through transmembrane domains. Covalent cysteine-based dimer formation has been extensively described for neurotransmitter transporters, such as the dopamine transporter, DAT [55] and the glycine transporter [56] as well as receptors [57]. The fact that dimeric forms of ZnT3 were resistant to reducing agents and increased in response to oxidative stress, lead us to investigate tyrosine-mediated dimerization. Since its discovery in 1959, dityrosine formation has been described as a post-translational modification related with cellular stress and disease [48]. Dityrosine modifications are produced in response to oxidative stress, aging [16], UV and γ irradiation [17]. Increased levels of dityrosine have been found in atheromatous plates [18], cataracts [16], acute inflammation, systemic bacterial infection [19] and recently associated with α-synuclein fibrillogenesis [20] and Aβ amyloid oligomerization [21]. Di-tyrosine formation as a normal post-translational modification has been described only in a limited group of structural proteins of the bacteria cell wall and insect ligaments [12], and in proteins of the extracellular matrix as collagen [13] and elastin [11]. Here we show tyrosine dimerization in a polytopic transmembrane protein, mediated by tyrosine residues in the carboxy terminal domain. In contrast to the described damage connotation and structural roles of dityrosine bonds, ZnT3 tyrosine modification presents a new functional paradigm for dityrosine bonds as regulators of both subcellular localization and metal transport activity. This ZnT3 post-translational modification occurs spontaneously and it is regulated by oxidative stress.

PC12 cells expressing *wild type* human ZnT3 and tyrosine mutants incubated with H_2_O_2_ enabled us to identify tyrosine 372 as a major residue mediating ZnT3 dimerization (Fig. 3). ZnT3 dimers formed between Y330-Y330, Y357-Y357 or Y330-Y357 are likely less abundant (Fig. 9, dotted line in Y372F mutant). Moreover, double mutants Y330F, Y357F containing an intact Y372, shows reduce dimerization. This indicates that Y372 formed dimers with Y330 or Y357 but not Y372-Y372 dimers (Fig. 9, Y300, 357F). Additionally, double mutant Y357F, Y372F, expressing an intact Y330 also shows reduce dimerization indicating that dimers Y330-Y330 are poorly represented (Fig. 9 Y357, 372F). The fact that no detectable phenotype was observed in Y330 mutant indicates that dimerization between Y372 and Y357 is the predominant state of ZnT3 dimers at steady state (Fig. 9 *wild type*).

Interestingly, the gain-of-function phenotype obtained by mutagenesis of tyrosine 357 in human ZnT3 indicates that tyrosine 357 might regulate the availability or proximity of Y330 and Y372 for dimer formation. Tyrosine residues involved in dityrosine bond formation or those adjacent could be subject to tyrosine post-translational modifications that could modulate dimerization and function of membrane proteins. For example NetPhos 2.0 (Technical University of Denmark) predicts that tyrosine 357 in human ZnT3, possesses a high probability for being a phosphate acceptor (0.633). Additionally, nitrosylation of tyrosines, a mofidication induced by oxidative stress, could modulate dityrosine bond formation of zinc transporters. These posttranslational events could affect tyrosine-dependent covalent dimerization, zinc transport, and cell response to metal challenges.

Zinc transport capacity is modulated by the dimeric state of ZnT3. Two lines of evidence lead us to conclude that dimers or higher oligomeric forms are the likely ZnT3 functional states. First, we observed a correlation between zinc transport into vesicle compartments and dimerization when comparing mouse and human ZnT3. The mouse ZnT3 fails to transport zinc into vesicle compartments as previously reported [23], [40]. In contrast, zinc transport capacity was 2.4 times higher in human ZnT3. Human ZnT3 forms dimers in response to H_2_O_2_, while the mouse transporter was almost insensitive. Moreover, we observed a significant zinquin staining in cells expressing ZnT4 and 5 but not ZnT7 (Fig. 5A). ZnT4 and ZnT5 form covalent oligomeric species in response to oxidative stress but not ZnT7 (Fig. 7B). Second, zinquin staining was lost in cells expressing the “loss-of-function” Y372F mutant (Fig. 5 C and D). Interestingly, zinc transport was not increased in the gain of function ZnT3Y357F mutant in assays that acutely assess zinc transport capacity (∼1 hour; Fig. 5 C and D), but became evident as a small yet significant increase in assays that explored sustained zinc transport capacity required to evade zinc toxicity (∼24 hours, Fig. 5E). In contrast, the loss-of-function ZnT3Y372F mutant increases cell death induced by zinc. Although, the mechanism is unknown, one possibility is that ZnT3Y372F may act as dominant negative mutant over endogenous zinc transporters leading to an unbalance of zinc homeostasis.

We described tyrosine mediated dimerization in two member of the SLC30A family, ZnT3 and ZnT4. Considering the distribution of charged residues surrounding ZnT3 and ZnT4 tyrosines, we propose that a putative motif YXXE is at least one of the primary sequences involved in dityrosine bond formation in ZnT family members. Whether this motif could be expanded to YXX[D/E] or to YXE remains unknown, yet these primary structure arrangements would encompass ZnT2 and ZnT1 as candidates for dityrosine bonding, respectively (Fig. 7A). The generality of a YXXE motif, whether this motif is an obligated component in dityrosine bond formation, or whether there are multiple sequences that could mediate dityrosine bond formation requires further testing in other membrane proteins.

Primary sequence determinants are not the only factors defining dityrosine-mediated dimerization. Differences in oligomerization and zinc transport capacity exhibited by the mouse and human ZnT3 orthologs pointed to a role of the amino terminal domain modulating dityrosine dimerization. Human ZnT3 amino terminal confers an enhanced capacity to respond to oxidative stress induced-oligomerization to mouse ZnT3. In contrast, mouse amino terminal confers an attenuated response to oxidative stress to the human transporter. These observations suggest species differences in biological mechanisms that depend on synaptic vesicle zinc content.

In conclusion we identified tyrosine dimerization as a new redox-sensitive post-translational modification in the SLC30A family of zinc transporters. Moreover, we demonstrate that dimers/oligomers are likely a functional intermediary in ZnT3 zinc transport. This mechanism uncovers new avenues of zinc homeostasis regulation by oxidative stress shared by several members of the SLC30A family. Elucidation of such mechanism will help us to understand the role of zinc and zinc transporters in pathologic conditions such as neurodegenerative diseases and diabetes, in which zinc transporters are thought to be involved.

## Supporting Information

Figure S1 Covalently modified ZnT3 is not sumoylated. PC12 cells non-transfected (mock) or transfected with HA-tagged versions of SUMO1, SUMO2 or SUMO3 were incubated without (A) or with H2O2 or MG132 (B). Triton-X100 soluble supernatant (500 Î¼g) were immunoprecipitated with HA antibodies and immunocomplexes analyzed by immunoblot with antibodies against either the HA epitope present in recombinant SUMO or myc engineered in ZnT3. HA immunoprecipitation did not isolated ZnT3 dimers or high molecular weight species under any condition. Unspecific binding of ZnT3 to HA-coated beads was detected in untransfected mock extracts (Fig. 3B lane 3). Input 2%.
(3.78 MB TIF)

## References

[1] Sitte HH, Farhan H, Javitch JA (2004). Sodium-dependent neurotransmitter transporters: oligomerization as a determinant of transporter function and trafficking.. Mol Interv.

[2] Chen X, Molino C, Liu L, Gumbiner BM (2007). Structural elements necessary for oligomerization, trafficking, and cell sorting function of paraxial protocadherin.. J Biol Chem.

[3] Pin JP, Kniazeff J, Liu J, Binet V, Goudet C (2005). Allosteric functioning of dimeric class C G-protein-coupled receptors.. Febs J.

[4] Nufer O, Kappeler F, Guldbrandsen S, Hauri HP (2003). ER export of ERGIC-53 is controlled by cooperation of targeting determinants in all three of its domains.. J Cell Sci.

[5] Robbins MJ, Calver AR, Filippov AK, Hirst WD, Russell RB (2001). GABA(B2) is essential for g-protein coupling of the GABA(B) receptor heterodimer.. J Neurosci.

[6] Baneres JL, Parello J (2003). Structure-based analysis of GPCR function: evidence for a novel pentameric assembly between the dimeric leukotriene B4 receptor BLT1 and the G-protein.. J Mol Biol.

[7] Funami K, Sasai M, Oshiumi H, Seya T, Matsumoto M (2008). Homo-oligomerization is essential for Toll/interleukin-1 receptor domain-containing adaptor molecule-1-mediated NF-kappaB and interferon regulatory factor-3 activation.. J Biol Chem.

[8] Lemmon MA, Flanagan JM, Treutlein HR, Zhang J, Engelman DM (1992). Sequence specificity in the dimerization of transmembrane alpha-helices.. Biochemistry.

[9] Senes A, Engel DE, DeGrado WF (2004). Folding of helical membrane proteins: the role of polar, GxxxG-like and proline motifs.. Curr Opin Struct Biol.

[10] Damian M, Martin A, Mesnier D, Pin JP, Baneres JL (2006). Asymmetric conformational changes in a GPCR dimer controlled by G-proteins.. Embo J.

[11] LaBella F, Keeley F, Vivian S, Thornhill D (1967). Evidence for dityrosine in elastin.. Biochem Biophys Res Commun.

[12] Andersen SO (1964). The Cross-Links in Resilin Identified as Dityrosine and Trityrosine.. Biochim Biophys Acta.

[13] LaBella F, Waykole P, Queen G (1968). Formation of insoluble gels and dityrosine by the action of peroxidase on soluble collagens.. Biochem Biophys Res Commun.

[14] Edens WA, Sharling L, Cheng G, Shapira R, Kinkade JM (2001). Tyrosine cross-linking of extracellular matrix is catalyzed by Duox, a multidomain oxidase/peroxidase with homology to the phagocyte oxidase subunit gp91phox.. J Cell Biol.

[15] Abdalla S, Lother H, El Missiry A, Sergeev P, Langer A (2008). Dominant-negative AT2 receptor oligomers induce G-protein arrest and symptoms of neurodegeneration.. J Biol Chem.

[16] Garcia-Castineiras S, Dillon J, Spector A (1978). Detection of bityrosine in cataractous human lens protein.. Science.

[17] Giulivi C, Davies KJ (1994). Dityrosine: a marker for oxidatively modified proteins and selective proteolysis.. Methods Enzymol.

[18] Hensley K, Maidt ML, Yu Z, Sang H, Markesbery WR (1998). Electrochemical analysis of protein nitrotyrosine and dityrosine in the Alzheimer brain indicates region-specific accumulation.. J Neurosci.

[19] Heinecke JW (2002). Oxidized amino acids: culprits in human atherosclerosis and indicators of oxidative stress.. Free Radic Biol Med.

[20] Krishnan S, Chi EY, Wood SJ, Kendrick BS, Li C (2003). Oxidative dimer formation is the critical rate-limiting step for Parkinson's disease alpha-synuclein fibrillogenesis.. Biochemistry.

[21] Atwood CS, Perry G, Zeng H, Kato Y, Jones WD (2004). Copper mediates dityrosine cross-linking of Alzheimer's amyloid-beta.. Biochemistry.

[22] Palmiter RD, Huang L (2004). Efflux and compartmentalization of zinc by members of the SLC30 family of solute carriers.. Pflugers Arch.

[23] Palmiter RD, Cole TB, Quaife CJ, Findley SD (1996). ZnT-3, a putative transporter of zinc into synaptic vesicles.. Proc Natl Acad Sci U S A.

[24] Cole TB, Wenzel HJ, Kafer KE, Schwartzkroin PA, Palmiter RD (1999). Elimination of zinc from synaptic vesicles in the intact mouse brain by disruption of the ZnT3 gene.. Proc Natl Acad Sci U S A.

[25] Kantheti P, Qiao X, Diaz ME, Peden AA, Meyer GE (1998). Mutation in AP-3 delta in the mocha mouse links endosomal transport to storage deficiency in platelets, melanosomes, and synaptic vesicles.. Neuron.

[26] Salazar G, Love R, Werner E, Doucette MM, Cheng S (2004). The zinc transporter ZnT3 interacts with AP-3 and it is preferentially targeted to a distinct synaptic vesicle subpopulation.. Mol Biol Cell.

[27] Dutzler R (2004). Structural basis for ion conduction and gating in ClC chloride channels.. FEBS Lett.

[28] Palmiter RD, Findley SD (1995). Cloning and functional characterization of a mammalian zinc transporter that confers resistance to zinc.. Embo J.

[29] Ellis CD, Macdiarmid CW, Eide DJ (2005). Heteromeric protein complexes mediate zinc transport into the secretory pathway of eukaryotic cells.. J Biol Chem.

[30] Suzuki T, Ishihara K, Migaki H, Matsuura W, Kohda A (2005). Zinc transporters, ZnT5 and ZnT7, are required for the activation of alkaline phosphatases, zinc-requiring enzymes that are glycosylphosphatidylinositol-anchored to the cytoplasmic membrane.. J Biol Chem.

[31] Suzuki T, Ishihara K, Migaki H, Nagao M, Yamaguchi-Iwai Y (2005). Two different zinc transport complexes of cation diffusion facilitator proteins localized in the secretory pathway operate to activate alkaline phosphatases in vertebrate cells.. J Biol Chem.

[32] Murgia C, Devirgiliis C, Mancini E, Donadel G, Zalewski P (2008). Diabetes-linked zinc transporter ZnT8 is a homodimeric protein expressed by distinct rodent endocrine cell types in the pancreas and other glands.. Nutr Metab Cardiovasc Dis.

[33] Lu M, Fu D (2007). Structure of the zinc transporter YiiP.. Science.

[34] Guo J, Wenk MR, Pellegrini L, Onofri F, Benfenati F (2003). Phosphatidylinositol 4-kinase type IIalpha is responsible for the phosphatidylinositol 4-kinase activity associated with synaptic vesicles.. Proc Natl Acad Sci U S A.

[35] Advani RJ, Yang B, Prekeris R, Lee KC, Klumperman J (1999). VAMP-7 mediates vesicular transport from endosomes to lysosomes.. J Cell Biol.

[36] Salazar G, Love R, Styers ML, Werner E, Peden A (2004). AP-3-dependent mechanisms control the targeting of a chloride channel (ClC-3) in neuronal and non-neuronal cells.. J Biol Chem.

[37] Falcon-Perez JM, Dell'Angelica EC (2007). Zinc transporter 2 (SLC30A2) can suppress the vesicular zinc defect of adaptor protein 3-depleted fibroblasts by promoting zinc accumulation in lysosomes.. Exp Cell Res.

[38] Craige B, Salazar G, Faundez V (2008). Phosphatidylinositol-4-kinase type II alpha contains an AP-3-sorting motif and a kinase domain that are both required for endosome traffic.. Mol Biol Cell.

[39] Clift-O'Grady L, Desnos C, Lichtenstein Y, Faundez V, Horng JT (1998). Reconstitution of synaptic vesicle biogenesis from PC12 cell membranes.. Methods.

[40] Salazar G, Craige B, Love R, Kalman D, Faundez V (2005). Vglut1 and ZnT3 co-targeting mechanisms regulate vesicular zinc stores in PC12 cells.. J Cell Sci.

[41] Harrison RW, Chatterjee D, Weber IT (1995). Analysis of six protein structures predicted by comparative modeling techniques.. Proteins.

[42] Harrison R (1999). A self-assembling neural network for modeling polymers.. J Math Chem.

[43] Bagossi P, Zahuczky G, J. T, I.T. W, R.W. H (1999). “Improved Parameters for Generating Partial Charges: Correlation with Observed Dipole Moments”.. J Mol Model.

[44] Harrison RW (2003). Amortized fast multipole algorithm for molecular modeling.. Proceedings of the International Conference on Computer Science and its Applications, Eds, Pradip Peter Dey, Mohammad N Amin, Thomas M Gatton ICCSA.

[45] DeLano WL (2002). The PyMOL Molecular graphics System.

[46] Saitoh H, Hinchey J (2000). Functional heterogeneity of small ubiquitin-related protein modifiers SUMO-1 versus SUMO-2/3.. J Biol Chem.

[47] Lee DH, Goldberg AL (1998). Proteasome inhibitors: valuable new tools for cell biologists.. Trends Cell Biol.

[48] Malencik DA, Anderson SR (2003). Dityrosine as a product of oxidative stress and fluorescent probe.. Amino Acids.

[49] Newell-Litwa KA, Salazar G, Smith Y, Faundez V (2009). Roles of BLOC1 and AP-3 complexes in cargo sorting to synaptic vesicles.. Mol Biol Cell In Press.

[50] Craige B, Salazar G, Faundez V (2004). Isolation of synaptic vesicles.. Curr Protoc Cell Biol Chapter.

[51] Grote E, Hao JC, Bennett MK, Kelly RB (1995). A targeting signal in VAMP regulating transport to synaptic vesicles.. Cell.

[52] Faundez V, Horng JT, Kelly RB (1997). ADP ribosylation factor 1 is required for synaptic vesicle budding in PC12 cells.. J Cell Biol.

[53] Schmidt A, Hannah MJ, Huttner WB (1997). Synaptic-like microvesicles of neuroendocrine cells originate from a novel compartment that is continuous with the plasma membrane and devoid of transferrin receptor.. J Cell Biol.

[54] Zalewski PD, Forbes IJ, Betts WH (1993). Correlation of apoptosis with change in intracellular labile Zn(II) using zinquin [(2-methyl-8-p-toluenesulphonamido-6-quinolyloxy)acetic acid], a new specific fluorescent probe for Zn(II).. Biochem J.

[55] Hastrup H, Karlin A, Javitch JA (2001). Symmetrical dimer of the human dopamine transporter revealed by cross-linking Cys-306 at the extracellular end of the sixth transmembrane segment.. Proc Natl Acad Sci U S A.

[56] Bartholomaus I, Milan-Lobo L, Nicke A, Dutertre S, Hastrup H (2008). Glycine transporter dimers: evidence for occurrence in the plasma membrane.. J Biol Chem.

[57] Tao RH, Maruyama IN (2008). All EGF(ErbB) receptors have preformed homo- and heterodimeric structures in living cells.. J Cell Sci.

