# Supplementary figures and images for: SLC30A3 (ZnT3) Oligomerization by Dityrosine Bonds Regulates Its Subcellular Localization and Metal Transport Capacity
Source: PLoS One. 2009 Jun 12;4(6):e5896. doi: 10.1371/journal.pone.0005896 (PMC2690824; doi:10.1371/journal.pone.0005896)

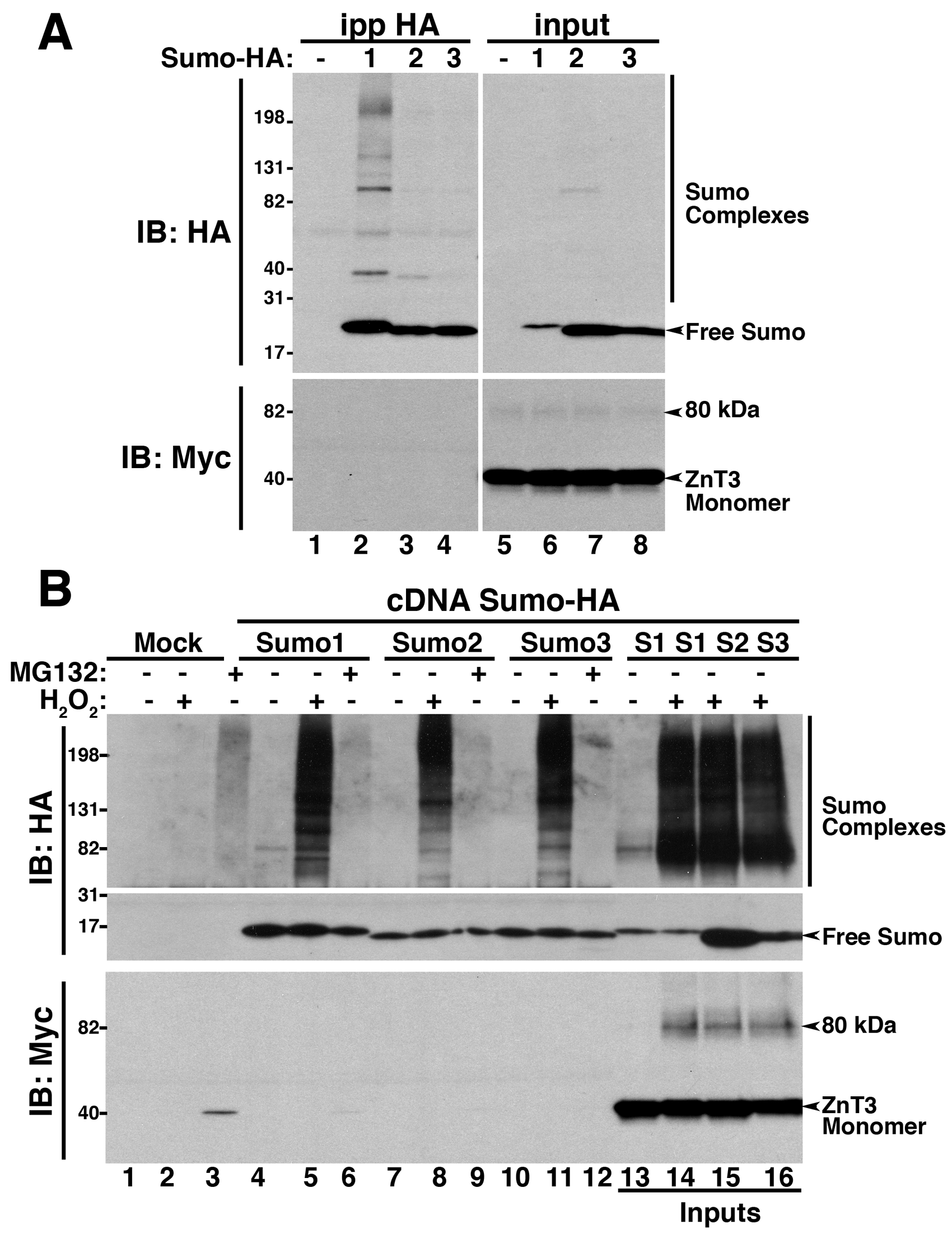

Supplement: Figure S1 — Covalently modified ZnT3 is not sumoylated. PC12 cells non-transfected (mock) or transfected with HA-tagged versions of SUMO1, SUMO2 or SUMO3 were incubated without (A) or with H2O2 or MG132 (B). Triton-X100 soluble supernatant (500 Î¼g) were immunoprecipitated with HA antibodies and immunocomplexes analyzed by immunoblot with antibodies against either the HA epitope present in recombinant SUMO or myc engineered in ZnT3. HA immunoprecipitation did not isolated ZnT3 dimers or high molecular weight species under any condition. Unspecific binding of ZnT3 to HA-coated beads was detected in untransfected mock extracts (Fig. 3B lane 3). Input 2%. (3.78 MB TIF) [file pone.0005896.s001.tif]
